# Innovative Green Technologies of Intensification for Valorization of Seafood and Their By-Products

**DOI:** 10.3390/md17120689

**Published:** 2019-12-06

**Authors:** Fadila Al Khawli, Mirian Pateiro, Rubén Domínguez, José M. Lorenzo, Patricia Gullón, Katerina Kousoulaki, Emilia Ferrer, Houda Berrada, Francisco J. Barba

**Affiliations:** 1 Department of Preventive Medicine and Public Health, Food Science, Toxicology and Forensic Medicine, Faculty of Pharmacy, Universitat de València, Avda. Vicent Andrés Estellés, s/n 46100 Burjassot, València, Spain; khawli@alumni.uv.es; 2 Centro Tecnológico de la Carne de Galicia, Rúa Galicia No 4, Parque Tecnológico de Galicia, San Cibrao das Viñas, 32900 Ourense, Spain; mirianpateiro@ceteca.net (M.P.); rubendominguez@ceteca.net (R.D.); patriciagullon@ceteca.net (P.G.); 3 Department of Nutrition and Feed Technology, Nofima AS, 5141 Bergen, Norway; katerina.kousoulaki@nofima.no

**Keywords:** high-added value compounds, seafood by-products, innovative green technologies, functional foods

## Abstract

The activities linked to the fishing sector generate substantial quantities of by-products, which are often discarded or used as low-value ingredients in animal feed. However, these marine by-products are a prominent potential good source of bioactive compounds, with important functional properties that can be isolated or up-concentrated, giving them an added value in higher end markets, as for instance nutraceuticals and cosmetics. This valorization of fish by-products has been boosted by the increasing awareness of consumers regarding the relationship between diet and health, demanding new fish products with enhanced nutritional and functional properties. To obtain fish by-product-derived biocompounds with good, functional and acceptable organoleptic properties, the selection of appropriate extraction methods for each bioactive ingredient is of the outmost importance. In this regard, over the last years, innovative alternative technologies of intensification, such as ultrasound-assisted extraction (UAE) and supercritical fluid extraction (SFE), have become an alternative to the conventional methods in the isolation of valuable compounds from fish and shellfish by-products. Innovative green technologies present great advantages to traditional methods, preserving and even enhancing the quality and the extraction efficiency, as well as minimizing functional properties’ losses of the bioactive compounds extracted from marine by-products. Besides their biological activities, bioactive compounds obtained by innovative alternative technologies can enhance several technological properties of food matrices, enabling their use as ingredients in novel foods. This review is focusing on analyzing the principles and the use of UAE and SFE as emerging technologies to valorize seafoods and their by-products.

## 1. Introduction

Fish is considered to be healthy, and to be among the most nutritious animal-derived foods, due to their content in a high quality of proteins, balanced essential amino acids, high levels of fat-soluble vitamins (A and D) and essential macro- and microminerals (iodine, magnesium, phosphorus and selenium) [1]. 

Moreover, marine fatty fish contain high levels of long chain highly unsaturated *n-3* fatty acids, which have been associated with reduction of the risk of cardiovascular diseases in humans [2]. Fish nutrient composition, mostly characterized by 15%–30% proteins, 0%–25% lipids and 50%–80% moisture, depends upon fish species, age, gender, health, nutritional status and time of the year. For instance, white fish such as cod and hake are lean species, containing ca. 20% protein, 80% water and rather low lipids levels (0.5%–3%), whereas fatty fish, such as mackerel and salmon, contain 20% protein, 10%–18% lipids, and correspondingly lower water content (62%–70%) [3].

In 2016, fish production worldwide amounted to ca. 171 million tons, 91 million tons deriving from inland and marine fisheries, and 80 million tons from aquaculture, with China being the largest producer [4]. In Europe, Norway and Spain are topping the list of the largest producing countries for capture fisheries (2.03 and 0.91 million of tons, respectively). As a consequence of the activities related to the different fishing sectors, a great amount of fish by-products, not utilized for direct human consumption, are generated every year, and they can represent anything betwen 30% and 85% of the weight of the different catches [5]. The food fish to by-product ratio varies by fishing zone, season, fish size and species [6]. Besides bycatch, fisheries and aquaculture by-products include fish fins, backbones, gills, heads, belly flaps, liver, roe, skin, viscera, among others [7]. Indicatively, heads represent 9 %–12%, viscera 12 %–18%, skin 1 %–3%, bones 9 %–15% and scales ca. 5 % of whole fish weight [8]. 

Fish by-products can entail significant environmental and food-technical challenges due to their high microbial and endogenous enzyme load, rendering them susceptible to rapid degradation if not processed properly or stored in appropriate conditions [9,10]. Fish by-products can be classified into two types: One that includes easily degradable products with high enzyme content, such as viscera and blood, and a second one that includes the more stable products (bones, heads and skin) [5]. Timely collection and the treatment of fish by-products is a crucial step in maintaining their quality to be used as raw materials for obtaining high added-value products [5] Given that fish production, landing and processing locations are spread geographically, it appears that the best management option that would allow the conversion of fish residues into products of greater value is that of processing locally immediately after production [11]. To achieve this, significant investments, for instance, on board fishing vessels, would be required, not easy to justify unless already developed markets for the new end-products are present. By refining seafood by-products, high-added value components for the production of nutraceuticals and bioactive ingredients can be obtained. Processing fish proteins can generate bioactive peptides, amino acids and other bioactive nitrogenous compounds [12], whereas fish oil by-products generated from a fish oil refinery can be utilized as raw materials for the production of the essential long chain, polyunsaturated fatty acids concentrates, eicosapentaenoic acid (EPA) and docosahexaenoic acid (DHA), to be used in food supplements [13]. 

To succeed in utilizing marine resources in a responsible and good way, it is indispensable to establish efficient and safe methods for the extraction of the target nutrients and bioactives. Downstream processing in the biomass refinery includes, among others, conventional techniques, already widely used for the separation, selective upconcentration and extraction of target compounds, such as in fish meal and fish oil [14] or EPA- and DHA-rich oil production [15]. These methods are efficient, and their main drawback is related to the high energy consumption and potential thermal degradation of target compounds, due to the high processing temperatures. Other extraction methods involving the use of organic solvents would entail risks for human health and the environment, and may also lead to perishable compound degradation, should prolonged extraction periods be involved [16].

In recent years, the concept of green technology, assuming the use of more environmentally-friendly techniques for ingredient processing, has emerged [17]. Innovative alternative extraction technologies, such as supercritical fluid extraction (SFE), ultrasound-assisted extraction (UAE), pulsed electric fields (PEF) or microwave-assisted extraction (MAE), have been identified as green extraction techniques for the separation of high-added value compounds [18,19]. 

These alternative technologies have several advantages, including rapid extraction, low solvent consumption rates, use of alternative environmentally-friendly solvents, superior compound recovery rates and higher selectivity.

This review intends to summarize the potential applications of UAE and SFE, as green technologies, for the extraction of a wide range of bioactive compounds from fish side stream biomasses, and thus achieve the valorization of seafood and their by-products. Moreover, this review also aims to provide detailed information on the potential benefits of applying these innovative technologies for a by-product refinery in both academy and the industry.

## 2. Valorization of Fish By-Products

**Figure 1:**
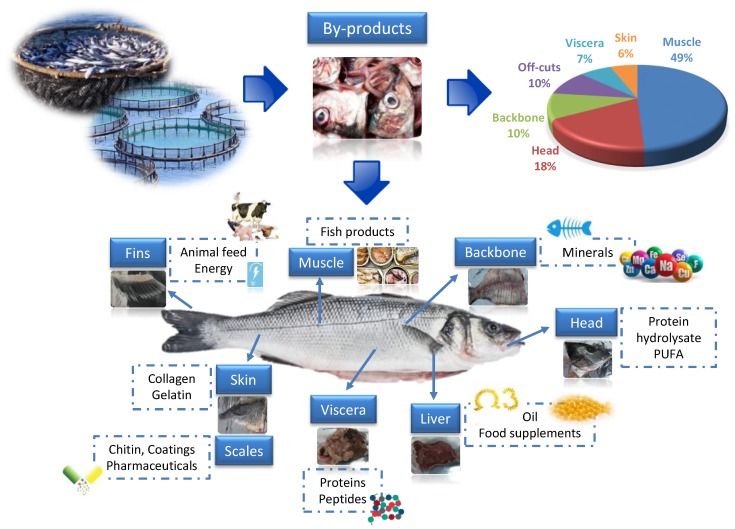
Fish processing by-product generation and end use opportunities.

There are multiple possibilities in valorizing marine by-products through processing, as for instance creating more valuable ingredients or extracting specific high-value compounds. Following the European Union (EU) Directive 2008/98/CE, a standard prioritization scheme can be established, visualized by a pyramid in which the obtained product value, as well as the necessary quality of the raw material used, decrease from top to bottom [20]. The main aspect in the model for marine biomass valorization is linked to the application of good practices, and therefore the prevention or reduction of wastes. Millions of tons of captured fish are returned to the sea for failing to comply with regulations regarding legal size, no control over catch rates, or low quality. This forced the European Union to establish a new fisheries’ policy that involves actions paving the way towards zero-discards [21]. To meet the goals set by the new policy, novel management measures must be established enabling the valorization of fish side stream biomasses. Maintaining marine catch discards and by-products in the food chain can be practiced either through the commercialization of low-value fractions, or through the production of ingredients and high-value biomolecules that can be used in the pharmaceutical and nutraceutical industry [22,23,24,25], fulfilling the principles of a sustainable circular economy (green approach). This complementary approach allows an efficient use of fish by-products, transforming them into ingredients that can be incorporated into feed, food or other high-value products (Figure 1). Use of fish by-products in animal feeds (flours and oils), is the most common option practiced today [26,27]. Finally, waste from the above processes may also have the potential to be used in biofuel production or be exploited in other agronomic and industrial applications, as for instance fertilizers [28,29].

The known healthy compounds and properties associated with fish are also present in their by-products. A great number of bioactive compounds can be obtained from fish by-products [11,13,30,31,32]: collagen [33], chitin [34], enzymes [35], gelatin [36], glycosaminoglycans [37,38], polyunsaturated fatty acids (PUFA) [39], minerals [40,41], protein and peptides [10,42,43] and vitamins. It should be noted that the long-chain omega-3 fatty acids (LC-PUFAs), eicosapentaenoic acid (EPA) and docosahexaenoic acid (DHA), are among the most successful compounds extracted from fish by-products, achieving a high value in the market due to their beneficial health effects [11]. Marine by-product-derived compounds are known to induce positive effects on human health associated with their, e.g., anticancer, antidepressant, anti-diabetic, antihyperglycemic, antihypertensive, anti-inflammatory, antimicrobial, antioxidant, antiproliferative, anti-rheumatoid and immunomodulatory properties [42,43,44]. Besides their biological activities exploited by pharmaceutical, nutraceutical and cosmeceutical industries [45], marine by-product ingredients can also provide desirable technological properties when included in food products, acting for instance as emulsifying and foaming agents, and facilitating fat binding, solubility and water holding capacity [46,47]. Recent data show that it is possible to modify fish burger technical properties, in terms of hardness, cohesiveness, juiciness and adhesiveness, by the addition of low amounts fish by-product protein powder or fish hydrolysates [48].

## 3. Emerging Technologies for the Extraction of Bioactive Compounds from Fishery By-Products

Several techniques can be used to extract bioactive compounds, thus valorizing fish by-products. Among the conventional methods that are used for the extraction of fishery by-products. it is possible to highlight enzymatic hydrolysis for the solubilization and upconcentration of fish proteins, as reviewed by Aspevik et al. (2017), and among others, lipid extracton by Soxhlet, steam distillation and the use of solvents. Some traditional extraction methods, besides being characterized by low extraction yields, long extraction time, high solvent and high energy consumption and potential health hazards [16], involve extraction conditions (pH, temperature, extraction time, solvent type, concentration, etc.) that can alter the functional properties of potentially valuable compounds. Therefore, there is a need to explore alternative processing technologies that can better preserve target bioactive components [49,50], operating at lower temperatures and avoiding as much as possible the use of solvents. The shortcomings of these conventional methods have stimulated the interest in emerging green technologies. Several techniques, such as PEF, UAE, MAE, SFE and high pressure can be used to extract bioactive compounds, thus valorizing fish by-products [51,52]. Among these innovative, alternative techniques are ultrasounds-assisted (UAE) and supercritical fluid extraction (SFE), which are the object of the present review.

### 3.1. Ultrasound-assisted Extraction (UAE)

#### 3.1.1. Fundamentals

The use of ultrasound has increased, and has been applied over the last years with the scope to minimize processing, maximize the quality and ensure the safety of food products. This technique is applied in improving the technological properties of food, such as emulsification ability, solubility and texture, as well as on applications such as preservation, homogenization, viscosity alteration, extraction, drying, crystallization and antifoaming actions and enzymatic activation and inactivation [53]. Nowadays, improvements in ultrasound technology grant the opportunity to extract bioactive compounds with economic advantages, and this is referred to as innovative UAE [53].

Ultrasound works in frequencies above human hearing levels, ranging from 20 kHz to 10 MHz [53], and is classified by the amount of energy generated as sound power (W), sound intensity (W/m^2^), or sound power density (W/m^3^). The use of ultrasounds can be divided into two types: high intensity and low intensity. Low-intensity ultrasounds with high frequency (100 kHz to 1 MHz), and low-power < 1 W/cm^2^ are used as non-destructive methods for evaluating the physical and chemical properties in food products [54], whereas high-intensity ultrasounds have low frequency (20 kHz-100 kHz) and high power >1 W/cm^2^, and are used to speed up and improve the efficiency of sample preparation, as they can alter the physical or chemical properties of food [54]. 

**Figure 2:**
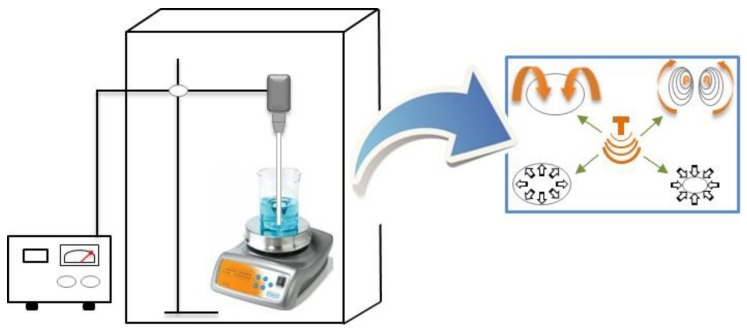
Schematic representation of the ultrasound-assisted extraction (UAE) process and the bubble cavitation phenomenon involved in this extraction technique.

UAE is generally recognized as an effective tool used in extraction methods, significantly minimizing the time required to increase both the productivity and the quality of the product. Numerous studies have critically assessed a variety of UAE applications in the industrial extraction of bioactive compounds [53] and found that this extraction technique enhances the yield of extraction, improving simultaneously their functional properties [55]. UAE efficiency is driven by the creation of acoustic cavitation and mechanical impact in the material matrix (Figure 2). Acoustic cavitation when used in plant materials can disrupt cell walls facilitating the solvent penetration into the sample matrix. Ultrasound mechanical impact increases the surface area of contact between the solvent and the extractable compounds, and hence offers greater penetration of solvents into the sample matrix, releasing in this way the bioactive compounds [53,56]. The UAE requires less extraction time and reduced solvent consumption. It can be performed at low temperatures, which can decrease the damages caused by temperature, and reduce the loss of bioactive substances [53]. In contrast, a denaturation of the protein/enzyme can occur when UAE is applied for a long period of time, since it results in high pressures, shear strength and increased temperatures into the medium.

#### 3.1.2. Use of UAE in Fish Industry

The utilization of ultrasound technology in the food industry is not new. Recently, UAE became recognized as an efficient, rapid, clean, reproducible and alternative non-thermal extraction technique as compared to conventional extraction methods [53]. Table 1 lists the advantages and drawbacks of the employment of UAE in marine products and discards. The application of UAE results in both the disruption of the material cell structures and an increase in the accessibility of the solvent to the internal particle structure, which enhances the intra-particle diffusivity. Hence, with significant improvements in both the extraction yield and time used, improved efficiency could be achieved when the substrate particle size is reduced [57].

In the last decades, researchers have reported that the optimization of several parameters, as for instance ultrasound frequency, propagation cycle (continuous or discontinuous), nominal power of the device, amplitude, type and the geometry of the system (e.g., length and diameter of the probe), improve the efficiency of UAE towards the extraction of target compounds [58]. Currently, UAE is widely used for the recovery of several valuable compounds from seafood by-products (Table 2) [54].

For instance, several studies reveal that UAE can be used successfully for collagen extraction from fish by-products (skin and scales), reducing processing time and increasing yield [59,60]. In the processing skin of Japanese sea bass (*Lateolabrax japonicus*) for the extraction of collagen using UAE, it was shown that the extraction yield differed according to the amount of acid added, the treatment time and the amplitude of the ultrasonic waves [60]. More in detail, when the treatment time was increased for a long period (24h), unknown components were obtained, most probably deriving from a breakdown of collagen, and conducting further optimization trials determined the most effective conditions for the extraction of pure collagen using USE (80% amplitude with 0.1 M acetic acid for 3 h of treatment).

Another important peptide for its emulsifying, foaming and gelling properties, is gelatin [61]. Gelatine is a polypeptide, which results from the denaturation of insoluble collagen, shown to have valuable functional properties, such as emulsifying, foaming, gelling, fat binding and water holding capacity [62]. Although the most widely used gelatins are of mammalian origin, the appearance of bovine spongiform encephalopathy (BSE, or mad cow) disease and religious restrictions regarding the consumption of porcine and bovine products, places marine collagen in a favourable position, rendering it as the most important alternative source. Several studies report the potential of using fish by-products, especially skins and bones, as novel sources of marine gelatin [32]. Limiting factors for the large-scale development of the fish gelatin industry are its inferior rheological properties, the lack of sufficient available raw materials and the variable quality of marine gelatin. In addition, other intrinsic quality factors related to odor, color, bloom strength and the viscosity of fish gelatin also limit the use of this gelatin [62]. 

In a study using the scales of bighead carp (*Hypophthalmichthys nobilis*), UAE (200 w, 60 °C, different extraction times from 1 h to 5 h) allowed an increase in extraction yields (30.94–46.67%) and the quality of the gelatin obtained as compared to using a water bath [63]. The authors reported that the extraction yields obtained with an ultrasound bath at 60 °C (46.67%) was also higher than those obtained with the water bath (36.39%) [63]. Furthermore, fish scales gelatins extracted with UAE are shown to have higher gelling and melting points, gel strength, apparent viscosity and emulsifying properties, compared to those obtained with a water bath extraction [59]. In another study, gelatin extracted by UAE was shown to have higher thermal stability compared with gelatin extracted by a conventional extraction. 

However, the application of a higher ultrasound intensity (over 200 W) and a more extended extraction time (above 5 h) can lead to the decrease in gel strength and melting points of gelatin, which may cause protein degradation due to acoustic cavitation [63].

Chitin, a polysaccharide present in the exoskeleton of crustaceans (shells) and the endoskeleton (pen) of cephalopods [67], is another compound that can be extracted with UAE. The influence of sonication time (0, 1 and 4 h) on yield, purity and crystallinity was evaluated during the extraction of chitin from North Atlantic shrimp (NAS) shells (*Pandalus borealis*). The investigation showed that the crystallinity indices and the extraction yield of chitin decreased as the sonication time increased (from 8.28% to 5.02% after 4 h of sonication treatments). Meanwhile, the extraction yield increased from 7.45% to 44.01% after 4 h of sonication treatment (Table 2) [65].

The combination of UAE with other technologies has also been studied in processing different fish by-products in order to improve the extraction efficiency and the quality of extracted bioactive compounds. In summary, pre-treatment with emerging technologies has the potential to increase the quality of the extracted compounds and thus their beneficial properties, as by using these techniques it is possible to nearly maintain their composition and structure intact.

Combining different novel technologies, such as UAE with enzymes, has also been demonstrated to improve extraction yields, facilitating an increase in collisions between enzyme and substrate [68]. Ultrasound-assisted enzymatic extraction is considered as a promising method for the improvement of the extraction yield of oil from marine matrices. Bruno et al. [64] evaluated the effects of pretreatments with UAE before enzymatic extraction on the extraction yield, fatty acid profile, oxidative stability and rheological properties of oil extracted from *Labeo rohita* heads (Table 2). The results showed higher oil recoveries, higher PUFA contents and higher oxidative stability in the samples subjected to a pretreatment with UAE before enzymatic hydrolysis. Besides, lower apparent viscosity and sensitivity to temperature changes were observed in the oil extracted using both UAE and enzymes as compared to enzymes alone [64].

In addition, Álvarez et al. [66] investigated the influence of UAE in the protein extraction yield from mackerel by-products by isoelectric solubilization precipitation (ISP). ISP is an emerging technology that uses pH changes to promote protein extraction. Several parameters influence the yield of extraction using this technology, such as the raw material quality as well as the extraction conditions (pH, temperature and extraction time). It was reported that by applying 60% of amplitude for 10 min in 0.1 M NaOH solution it was possible to recover ≈94% of total raw material protein in a single extraction step. It was also shown that lower amplitudes (20%) of ultrasonic bath increases the yield of the extraction when compared to traditional ISP. Furthermore, applying UAE to alkaline extraction allowed the recovery of more than 95% of total protein from mackerel by-products [66]. Therefore, the use of UAE in combination with ISP for protein extraction from fish by-products can give higher yields, using lower extraction times and less solvent [69].

### 3.2. Supercritical Fluid Extraction (SFE)

#### 3.2.1. Fundamentals

**Figure 3:**
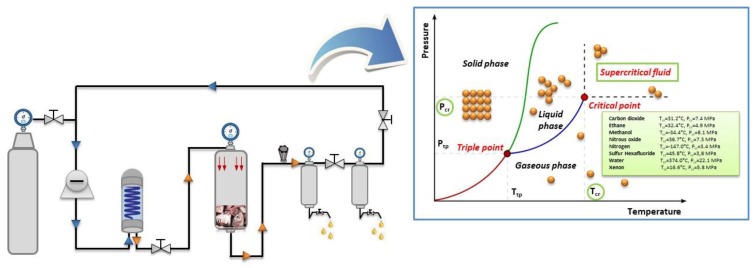
Schematic representation of supercritical fluid extraction (SFE) and the mechanism involved in this extraction technique.

SFE is an alternative extraction method that has attracted a growing attention in food industries in the last decade. It is considered a green technology due to the utilization of non-toxic organic solvents, which results in more sustainable processing and reduced energy use and environmental pollution (Table 1) [70]. In SFE, solvents are used at above or near their critical temperature and pressure to separate solutes from a liquid or solid matrix under pressurized conditions. Under these conditions the solvents have intermediary properties between gases and liquids, which facilitates the extraction of the target compounds (Figure 3). Carbone dioxide (CO_2_) is the most widely used SFE solvent in food applications, since it is generally recognized as safe (GRAS) [71]. CO_2_ is not only cheap and easily available at high purity, but also lacks toxicity and flammability. It has a moderate critical temperature and pressure (31.1 °C and 7.4 MPa), and can be readily removed by a simple pressure reduction [72]. Furthermore, its higher diffusion coefficient and lower viscosity allow the rapid penetration through the pores of heterogeneous matrices, like gas, helping to dissolve the solute like a liquid. The efficiency of the SFE process is mostly affected by pressure, extraction temperature, extraction time, CO_2_ density, CO_2_ flow rate and co-solvent concentration [73]. The SFE selectivity is achieved by adjusting temperature and pressure, resulting in alterations of the density. This selectivity can also be adjusted by the use of a co-solvent, either to increase or decrease the polarity of CO_2_. The most frequently used co-solvent is ethanol, because it meets the green technology requirements.

#### 3.2.2. Application of SFE in By-Products from Fish Industry

SFE has been used widely in several areas of food technology for food safety, food drying and sterilization, and food oil removal applications. This extraction technique is already being applied in the extraction of valuable compounds from natural materials, such as plant and marine sources. Several natural compounds, such as vitamins, flavors, natural pigments and essential oils, are extracted with SFE, thus avoiding the use of organic solvents and high temperatures [74]. So far, most of the studies that have evaluated the potential of SFE to extract biomolecules from fish by-products have focused upon lipid-soluble and antioxidant compounds [73,75]. Table 3 collects the advantages and drawbacks of the employment of SFE in processing marine products and discards. 

Nowadays, the large demand on fish oil by consumers linked to the large amount of fish by-products generated every year that are discarded has increased the interest regarding the extraction of edible fish oil from fish by-products (Table 4) using SFE. During SFE, the extraction parameters used (extraction time, flow-rate of CO_2_, pressure and temperature) play a key role on the extraction yield and the lipid composition of the functional products obtained. SFE has been applied to extract an oil fraction from fish meal. Fish meal is one of the primary products obtained from fish processing [76]. Its composition stands out for its higher protein content and balanced amino acid profile, characterized by good digestibility. Fish meals can be used to obtain fish protein concentrates intended for human consumption, as well as low-fat protein hydrolysates, thus achieving consumer demands for healthier fish products [77]. 

SFE allowed us to reduce the fat content of the produced fish meal without affecting protein quality. Extraction conditions of pressure (10–40 MPa), temperature (25–80 °C), and CO_2_ flow-rates of 9.5 g/min resulted in a product with a 90% reduction of fat and a lighter color, as with this method pigments such as astaxanthin were also extracted.

Moreover, SFE-extracted oils have also been shown to have higher radical scavenging activity and longer oxidative stability [84]. Using a gas saturated solution process, employing similar extraction conditions as that of SFE, in mackerel muscle, resulted in a more stable and less oxidized oil. However, the yields were low, obtaining oil concentrations of 4.00 g/20 g of mackerel muscle [83].

Longtail tuna (*Thunnus tonggol*) heads have also been used to obtain PUFA using SFE [80,81]. Tuna oil, besides omega-3 PUFA, also contains substantial levels of saturated fatty acids (SFA) and undesirable impurities which were extracted by simultaneous fractionation using SFE with ethanol as a co-solvent. In this process, fish oil was extracted and simultaneously collected into six fractions based on molecular weight. The short chain SFA fraction was extracted early, while the latter fractions were dominated by long-chain fatty acids, especially monounsaturated fatty acids (MUFA) and PUFA, particularly rich in DHA among other omega-3 and omega-6 fatty acids, resulting in a refined product with added value for health. The conditions that yielded optimal results in terms of obtaining a PUFA-rich fraction with a high quality and storage stability were 65 °C, 40 MPa, with a CO_2_ flow of 3.0 mL/min during 120 min. The results of this study demonstrate that, in applying SFE, the utilization of ethanol as the co-solvent allows us to achieve an upconcentration of PUFA (omega-3 and omega-6) in an effective way, and that using SFE for the extraction of fish oil from fish by-products can play an important role in obtaining economic and nutritional benefits, reducing environmental risks [84] (Table 4).

Sahena et al. compared different techniques for oil extraction from Indian mackerel (*Rastrelliger kanagurta*) skin [86]. Oil from this by-product fraction was extracted by SFE at different pressures (20–35 MPa) and temperatures (45–75 °C), and was compared to Soxhlet extraction [70,86]. The authors observed that their oil extraction yield increased with pressure and temperature, being 53.2% for SFE co-solvent, 52.8% for soaking pressure and 24.7% for the continuous technique at 35 MPa and 75 °C. The Soxhlet method achieved the highest extraction yield (53.6%) compared to that obtained with SFE. Other studies have demonstrated that the pressure swing and soaking techniques are among the most effective ones in extracting oil from fish skin [70,86]. 

Létisse et al. [44] also evaluated the influence of SFE conditions (pressure, temperature and CO_2_ rate) on the upconcentration of EPA and DHA in oil from sardine heads and tails. The obtained results confirmed that conditions of 30 MPa, 75 °C, 2.5 mL CO_2_/min and 45 min of extraction time allowed the obtaining of yields of 10.36%, and contents of EPA and DHA of 10.95% and 13.01%, respectively. Rubio-Rodríguez et al. [91] found that the application of lower pressure and temperature (25 MPa, 40 °C), higher CO_2_ flow (10 kg CO_2_/h) and an upflow direction through the offcuts from two hake species (*Merluccius capensis–Merluccius paradoxus*) during 3 h resulted in extracting more than 96% of the total oil contained in the raw material. High contents of EPA and DHA (about 6% and 14%, respectively, of the total fatty acids) were obtained in the extracted oil [91]. Furthermore, the application of the aforementioned conditions of temperature and pressure on the off-cuts of orange roughy (*Hoplostethus atlanticus*) and Atlantic salmon (*Salmo salar*), as well as on liver from jumbo squid (*Dosidicus gigas*) resulted in fish oils with reduced PUFA oxidation and less impurities [85]. The application of SFE in tuna livers also allowed to result in oil both rich in n-3 PUFA and vitamins [82].

Fish by-products, such as caviar and viscera, are also an important source of bioactive compounds, especially of monounsaturated (MUFA) and polyunsaturated (PUFA) fatty acids [79]. In the case of viscera, the application of conditions of 400 bar, 60 °C and a CO_2_ flow rate of 0.194 kg/h resulted in high yields (above 50 g/100g), which are similar to those obtained with petroleum ether, and the production of omega-enriched fish oils (DHA and EPA). Lisichkov et al. [90] studied the influence of operating parameters (pressure: 200, 300, 350 and 400 bar; temperatures: 40, 50 and 60 °C; CO_2_ flow rate: 0.194, 0.277 and 0.354 kg/h; and extraction time: 30, 60, 120 and 180 min) on the SFE extraction of PUFA from the viscera of common carp. For this purpose, authors used the 3D response surface methodology (RSM) and found that an equilibrium state was achieved after 180 min, where the curve of the extraction yield and the extraction time reached a plateau. 

The higher extraction yield was achieved at 180 min of extraction time, 60 °C of temperature, 400 bar of pressure and with a 0.354 kg/h CO_2_ flow rate. A positive impact of the increase of pressure and CO_2_ flow rate was observed on the extraction time and the total extraction yield, whereas the operating temperature had a complex influence, depending on the values of the operating pressure at isobaric conditions (Table 4) [90].

The yield and quality of oil extraction using different conventional versus emerging technologies were also evaluated by Fang et al. [82], who concluded that the best results were obtained using SFE and SC-dimethyl ether (SDEE), as these methods prevented the oxidation of lipids and reduced the damage of PUFA and vitamins, as compared with conventional methods (wet reduction and enzymatic extraction). Moreover, only a minor difference between the resulting material levels in volatile compounds and vitamins was observed in both SFE and SDEE, which was related to the used solvents’ solubility [82]. The disadvantages of SFE are related to high energy consumptions due to the application of high pressures and the need for material preparation by freeze-drying [82]. The limitation of SDEE is its lower critical point density and the related environment hazards [92]. Likewise, Taati et al. [78] found that SFE gives high extraction yields preventing oil oxidation, especially in oils with a high level of triacylglycerol (TAG) and PUFA, and attributed this result to the vacuum conditions and absence of free atmospheric oxygen during processing.

Finally, following extraction, the residues of fish by-products can also be used as a source of other valuable ingredients, such as amino acids, facilitated by the defattening amounts of the raw material which allows the extraction of other biomolecules [88]. Accordingly, Uddin et al. [88] evaluated the combined effect between SFE and sub-critical water hydrolysis (SWH) in order to obtain valuable materials from squid viscera. SWH is a technique considered as a non-conventional extraction method (green technology) that uses water in a sub-critical state as the solvent (from 100 ºC to 374 ºC at 0.10 MPa and 22 MPa, respectively). This enables the extraction of bioactive compounds of an ionic, polar and non-polar nature. This method has been used in several studies for the extraction of peptides and amino acids from animal by-products by hydrolyzing and breaking down the protein [93]. The results obtained in deoiled squid viscera confirmed that the use of SFE before SWH had positive effects on the recovery of amino acids, since the contents obtained in pretreated samples were 1.5 times higher than those obtained from raw squid viscera (51% *vs.* 76%, respectively).

The viscera of squid (*Todarodes pacificus*) was also processed to obtain other bioactive compounds such as enzymes and lecithin [87,89]. In the first case, *n*-hexane treatment of squid viscera resulted in the highest extraction yield; however, the thermal stability of digestive enzymes (protease, lipase and amylase) were slightly greater in SFE-treated samples [87]. High oxidative stability was also found in squid viscera lecithin subjected to a defattening step using SFE, despite its significant content in LC-PUFAs (EPA and DHA) [89].

#### 3.2.3. Application of SFE in by-Products from Processing Shellfish

Shellfish are marine organisms rich in several bioactive components with potential health benefits, which makes them interesting as functional food ingredients [12]. SFE has also been used to extract PUFAs from shrimp by-products (Table 5). Northern shrimp (*Pandalus borealis* Kreyer) processing by-products, such as heads, shell and tail could be used as a natural source for the development of beneficial health products (omega-3 PUFA) [94]. Depending on the extraction conditions used, different extraction yields and qualities can be obtained. The use of low pressure conditions (15 MPa and 50 °C) with flow rates of 3–5 L/min during 90 min showed high selectivity for DHA and EPA, while moderate pressures (35 MPa and 40 °C) showed increase extraction efficiency but lower yields than those obtained with organic solvents (137 mg oil/g *vs*. 206 mg oil/100 g and 178 mg oil/g, for SFE, acetone and *n*-hexane, respectively). In contrast, the obtained extract by SFE contained higher total free fatty acids (795 mg/g), and similar levels of EPA (7.8%) and DHA (8.0%) to conventional solvent extraction (Soxhlet using acetone and *n*-hexane as solvents), but with lower extraction times (90 min vs. 8 h, for SFE and Soxhlet extraction, respectively).

Recently, PUFA-rich lipids, in particular DHA and EPA, have been recovered with high yields (94% relative to the yield of Soxhlet extraction) from Rock lobster livers by SFE extraction [97]. Besides the use of this technique to obtain essential fatty acids for human consumption from this discard material, it also allowed us to reduce the presence of heavy metals in a product usually characterized by high contamination levels of arsenic and cadmium. This is due to the ability of SFE to carry out selective extraction of low-polar lipid compounds, retaining polar impurities such as some organic derivatives with heavy metals [85].

Another important compound that can be obtained from shellfish by-products is astaxanthin. As commented previously, astaxanthin is a pigment present in marine foods [99], such as fish (salmon and trout) and shellfish (shrimp and lobster). SFE is a selective and precise method that allows the extraction of astaxanthin from crustacean samples [95,98,100,101], achieving yields of total carotenoid extraction up to 98%, vs. 84% obtained with conventional extraction methods [100]. Depending on the extraction conditions, it is possible to achieve astaxanthin yields of about 40% [101]. Redspotted shrimp (*Farfantepenaeus paulensis*) heads, shells and tails are another source of astaxanthin, but the yields obtained by SFE in the published study by Sánchez-Camargo et al. (2011) were low [95]. The use of ethanol as co-solvent in different ratios improved the extraction of astaxanthin, as it allowed one to extract more than non-polar compounds [102], increasing the recoveries significantly (65.2% vs. 36%) [103]. Crawfish shell is also a source of astaxanthin. The application of similar SFE conditions (50 °C, 22.4 MPa, 1.0-1.5 L/min of CO_2_ flow rate, 10% of ethanol) to previously reviewed studies resulted in a significant increase of the extraction yield (197.6 mg/kg) [98].

## 5. Conclusions

There is a great increase in interest for the extraction of bioactive compounds from fish and shellfish by-products due to their nutritional value and potential health benefits. The valorization strategy of seafood by-products based on the development of novel products can lead to the more environmentally sustainable use of marine resources and higher economic benefits for the sector. It is thus critical to define appropriate extraction technologies that allow minimizing processing, maximizing quality and yield and ensuring product safety (non-toxic organic solvents) meeting thus the objectives for sustainable development in achieving food safety and food security for the increasing global human population. UAE and SFE are two emerging technologies that allow enhancing the extraction of thermolabile bioactive compounds, maintaining their quality and oxidative stability. Combining UAE and SFE with other extraction methods (ISP, SWH or enzymatic methods) can further increase extraction yields and reduce the presence of undesirable compounds (heavy metals). Finally, the use of UAE and SFE as a pretreatment to other methods offers the possibility of extracting even more valuable compounds from fish by-product matrices. 

## Figures and Tables

**Table 1:** Advantages and disadvantages of the application of ultrasound-assisted (UAE) extraction in fish and fish by-products for the extraction of bioactive compounds.

Extraction Technique	Advantages	Drawbacks	Extraction Conditions	Solvents
UAE	Reduction of energy, time and solvent consumption	Can induce lipid oxidation: increasing temperature by cavitation; formation of free radicals by sonolysis; mechanical forces generated by shockwaves and microstreaming.	25 kHz 200–2450 W 30-60 min	Ethanol, cyclohexane, other organic solvents
	Safe; does not produce toxic compounds	High power consumption		
	Higher penetration of solvent into cellular material and enhanced release in medium	Difficult to scale up		

**Table 2:** Bioactive compounds obtained from fish and shellfish by-products by UAE.

By-Product	Source	Bioactive Compound and Product	Extraction Conditions	Main Effects	Ref.
Head	*Labeo rohita*	Oil	UAE: 20 kHz, 40% amplitude, for 5, 10 and 15 min. Enzymatic hydrolysis: Protamex ratio of 1:100 (*w*/*w*), 2 h, 150 rpm, 55 °C.	Pretreatments with UAE improved the extraction yield of oil, showing higher oil recoveries (67.48% vs. 58.74 % for SFE and untreated samples, respectively).	[64]
Scales	Bighead carp (*Hypophthalmichthys nobilis*)	Gelatin	Temperature: 60, 70 and 80 °C Extraction time: 1 h	Improved technological properties: highest storage modulus (5000 Pa), gelation point (22.94 °C), and melting point (29.54 °C).	[59]
	Bighead carp (*Hypophthalmichthys nobilis*)	Gelatin	Temperature: 60 °C Extraction time: 1, 3 and 5 h	Extraction yield: 46.67% for ultrasound bath versus 36.39% for water bath.	[63]
Shells	Prawns (*Macrobrachium rosenbergii*)	Chitin	Extraction time: 0, 1, and 4 h 0.25M NaOH at solid to liquid ratio of 1:40 (*w*/*v*) Power: 41 W/cm.	Decrease of the crystallinity indices and extraction yield of chitin as the time of sonication increased.	[65]
Skin	Japanese sea bass (*Lateolabrax japonicus*)	Collagen	UAE: 20 kHz, 80% amplitude, 0.1 M acetic acid, 3 h.	UAE did not alter the major components of collagen (α1, α2 and β chains).	[60]
Whole fish	Mackerel	Proteins	ISP: Isoelectric solubilization precipitation. UAE: 40 kHz, 60% amplitude, 0.1 M NaOH, 10 min.	Significant increase of protein recovery, recovering more than 95% of total protein from mackerel by-products.	[66]

**Table 3:** Advantages and disadvantages of the application of supercritical fluid (SFE) extraction in fish and fish by-products for the extraction of bioactive compounds.

Extraction Technique	Advantages	Drawbacks	Extraction Conditions	Solvents
SFE	Green extraction Technique. No need for organic solvent, and therefore the extract is very pure. Lipids can be used immediately	Very expensive and complex equipment operating at elevated pressures	25–40 MPa 40–80 °C CO_2_ flow > 2 mL/min 45 min-6 h	Co-solvent: Ethanol
	Maintain the quality of the final product. Low operating temperatures (40–80 °C)	No polar substances are extracted		
	Free of heavy metals and inorganic salts	High power consumption		
	Very effective because of its low viscosity and high diffusivity. Fast and high yield			

**Table 4:** Bioactive compounds obtained from fish and fish by-products by SFE.

By-Product	Source	Bioactive Compound and Product	SC-CO_2_ Conditions		Outcomes	Ref.
Canned by-product	Tuna	Oils (volatiles)	Temperature ≥ 40 °C Pressure ≥ 25 MPa CO_2_ flow ≥ 10 kg/h Extraction time: 3 h		Extracted oils showed better conditions, quality (type of compounds and indicators of lipid oxidation) and yield.	[78]
Caviar, fillet and viscera	Carp (*Cyprinus carpio* L.)	Oil (MUFA and PUFA)	Temperature: 40, 50 and 60 °C Pressure: 200, 300, 350 and 400 bar CO_2_ flow: 0.194 kg/h Extraction time: 180 min		Omega-enriched fish oils (DHA and EPA). High yields, above 50 g/100 g in viscera, which are similar to those obtained with petroleum ether.	[79]
Fish meal	n.a. ^1^	Oil (MUFA and PUFA)	Temperature: 25–80 °C Pressure: 10–40 MPa CO_2_ flow with ethanol: 9.5 g/min		High reductions of fat (90%). Extract with a lighter colour due to astaxanthin extraction.	[77]
Head	*Thunnus tonggol*	Fatty acid (omega 3 and omega 6)	Temperature: 65 °C Pressure: 40 MPa CO_2_ flow with ethanol: 3 mL/min Extraction time: 2 h		SC-CO_2_ (co-solvent) is a good technique to extract omega3/6 after fractionations of oil.	[80]
		PUFA	Temperature: 65 °C Pressure: 40 MPa CO_2_ flow with ethanol: 2.4 mL/min Ethanol flow: 0.6 mL/min Extraction time: 120 min		Good quality of extracted PUFA-rich fraction, even 60 days after storage.	[81]
Heads and tails	Sardine	DHA and EPA	Temperature: 75 °C Pressure: 300 bar CO_2_ flow: 2.5 mL/min Extraction time: 45 min		Increase of the extraction yields: DHA (59%), EPA (28%).	[44]
Liver	Tuna	Fatty acids	Step of freeze-drying (12h) Temperature: 40 °C Pressure: 35 MPa Continuous CO_2_ flow: 3mL/min (at 20 °C) Extraction time: 4h		High quality and excellent yield obtained 98.45%.	[82]
Muscle	Mackerel	Vitamins	Temperature: 45 °C Pressure: 15–25 MPa CO_2_ flow: 27 g/min Extraction time: 2 h		High extraction of vitamins A, D2, D3 and α-tocopherol	[83]
Muscle, bone and skin	Salmon	Oil (PUFA)	Temperature: 45 °C Pressure: 250 bar CO2 Flow: 27g/min Extraction time: 3 h		Premium quality oil of physical, biochemical and biological properties. Yield 76.12 %–86.99%.	[84]
Muscle	Mackerel	Oil (EPA and DHA)	Temperature: 45 °C Pressure: 15–25 MPa CO_2_ flow: 27 g/min Extraction time: 2 h		The extracted oil presented significant contents of PUFAs (EPA, DHA). Higher stability compared with *n*-hexane extracted oil.	[83]
Off-cuts	Hake (*Merluccius capensis*– *Merluccius paradoxus*)	Oil (omega-3 fatty acids)	Temperature: 313 K Pressure: 25 MPa CO_2_ flow: 880 kg/m^3^		PUFA extraction. Reduction of fish oil oxidation. Reduction of certain impurities. Co-extraction of some endogenous volatile compounds.	[85]
	Orange roughy (*Hoplostethus atlanticus*)					
	Salmon (*Salmo salar*)					
Liver	Jumbo squid (*Dosidicus gigas*)					
**Skin**	Mackerel (*Rastrelliger kanagurta*)	Oil (PUFA)	Temperature: 45–75 °C Pressure: 20–35 MPa	Continuous: Pressurized (5 min, CO_2_ flow 2 mL/min	Yield very close to those obtained with the Soxhlet technique.	[86]
				Co-solvent technique: CO_2_ and ethanol (80%–20% at 2 mL/min) for 6 h	PUFA constituents of co-solvent, soaking and pressure swing techniques were similar to the Soxhlet method.	
				Soaking: Samples soaked with pure CO2 for 10 h then extracted for 6 h	The largest recoveries of PUFA, especially the ω-3 family, were achieved from the soaking and pressure swing techniques at 35 MPa and 75 °C.	
				Pressure swing: Samples pressurized (CO_2_) (2 h, extracted 3 h		
Viscera	Squid (*Todarodes pacificus*)	Enzymes	Temperature: 35–45 °C Pressure: 15–25 MPa CO_2_ flow: 22 g/min Extraction time: 2.5 h		Thermal stability of enzymes was slightly higher than *n*-hexane-treated squid viscera. Denaturation of proteins did not occur.	[87]
		Amino acids	SFE: Temperature: 35–45 °C Pressure: 15–25 MPa CO_2_ flow: 22 g/min Extraction time: 2.5 h	SWH: Temperature: 180–280 °C Pressure: 0.101–6.41 MPa Extraction time: 5 min	Positive effects of the use of SFE as a pretreatment method. Amino acids were 1.5 times higher than those obtained in non-deoiled samples.	[88]
		Lecithin	Temperature: 35–45 °C Pressure: 15–25 MPa CO_2_ flow: 22 g/min Extraction time: 2.5 h		Extraction yield was higher at the highest temperature and pressure (0.34 g/g squid viscera at 45 °C and 25 MPa). Lecithin that was isolated had in its composition some polyunsaturated fatty acids (EPA and DHA) with a high oxidative stability.	[89]
	Common carp (*Cyprinus carpio* L.)	PUFA	Temperature: 40, 50 and 60 °C Pressure: 200, 300, 350 and 400 bar CO_2_ mass flow: 0.194, 0.277 and 0.354 kg/h Extraction time: 30, 60, 120 and 180 min		Adequate for the isolation of bioactive components. Positive impact on the total yield and extraction time.	[90]

**Table 5:** Bioactive compounds obtained from shellfish by-products by supercritical fluid extraction (SFE).

By-Product	Source	Bioactive Compound	SC-CO_2_ Conditions	Outcomes	Ref.
Head, shells and tails	Brazilian redspotted shrimp (*Farfantepenaeus paulensis*)	Lipids and carotenoids	Temperature: 50 °C Pressure: 30 MPa CO_2_ flow: 4.2 × 10^−5^ kg/s Extraction time: 20 min Solvent for compounds recovery: *n*-hexane	Increase extraction yield: Astaxanthin (36%)	[95]
			Temperature: 50 °C Pressure: 30 MPa CO_2_ flow with ethanol: 8.3 × 10^−5^ kg/s Ethanol flow: 4.4 × 10^−6^ kg/s Extraction time: 200 min Solvent for compounds recovery: *n*-hexane	Increase extraction yield: Astaxanthin (57.9%)	
			Temperature: 43 °C Pressure: 370 bar CO_2_ flow: 1.5 L/min Extraction time: 200 min Solvent for compounds recovery: *n*-hexane	Increase extraction yield: Astaxanthin (39%)	[96]
	Northern shrimp (*Pandalus borealis* Kreyer)	PUFA	Temperature: 40 °C Pressure: 35 MPa CO_2_ flow: 3-5 L/min Extraction time: 90 min	Lower yields (137 mg oil/g) than those obtained in organic solvent extraction. Higher contents of total fatty acid content (795 mg/g), DHA (8%), EPA (7.8%).	[94]
Liver	Rock lobsters (*Jasus edwardsii*)	PUFA and vitamins	Temperature: 50 °C Pressure: 35 MPa Continuous CO_2_ flow: 0.434 kg/h Extraction time: 4h	Enrichment in PUFAs (DHA, EPA) vs. Soxhlet extraction. Reduction in the amounts of toxic heavy metals.	[97]
Shell	Crawfish	Pigments	Temperature: 50–70 °C Pressure: 13.8-31.0 MPa CO_2_ flow: 1.0–1.5 L/min Co-solvent: 10% ethanol	Increase extraction yield: Astaxanthin (197.6 mg/kg)	[98]
